# Sonographically Diagnosed Vault Hematomas Following Vaginal Hysterectomy and Its Correlation with Postoperative Morbidity

**DOI:** 10.1155/2007/91708

**Published:** 2007-02-28

**Authors:** Cem Dane, Banu Dane, Ahmet Cetin, Murat Yayla

**Affiliations:** Department of Gynecology and Obstetrics, Haseki Training and Research Hospital, 34019 Istanbul, Turkey

## Abstract

*Objective*. Our aim is to investigate sonographically detectable vault hematomas after vaginal hysterectomy and its relation to postoperative morbidity. *Methods*. We studied a group of 103 women who had undergone vaginal hysterectomy for benign causes apart from uterovaginal prolapse. Transabdominal ultrasound examinations were carried out 24 to 72 hours after surgery to assess the presence of vault hematomas. Ultrasound findings were correlated with clinical data and postoperative morbidity. *Results*. The incidence of vault hematoma was found 19.4% in present study. In these patients, 40% (8/20) had fever while only 2.4% (2/83) of cases without vault hematoma suffered from fever. Out of all women having vault hematoma, 70% (14/20) had small-sized hematoma and 30% (6/20) had large-sized hematoma. Fifty percent of patients (3/6) with large-sized hematoma, as compared to only 35% (5/14) with small-sized hematoma, suffered from febrile morbidity. Large-sized hematomas drained by vaginally, while all small-sized pelvic hematomas managed by watchful expectancy successfully. The significant difference was found mean hemoglobin drop and postoperative stay in the hematoma group or without hematoma group. *Conclusion*. Sonographic detection of vaginal vault fluid collection is common after hysterectomy, but such a finding rarely indicates additional treatment. Though febrile morbidity was more in cases with vault hematoma, the number of such patients was too small to be significant. Vaginal ultrasound examination should not be performed routinely after hysterectomy.

## 1. INTRODUCTION

Gynecologists for uterovaginal prolapse preferred vaginal hysterectomy where anterior colporrhaphy and posterior colpoperineorrhaphy can be conveniently performed. This route is associated with less febrile morbidity, less risk of hemorrhage, fewer blood transfusions, shorter hospitalization, and quick convalescence as compared to abdominal route [1–3]. Even with this procedure, some complications like hemorrhage, postoperative fever, and infection were reported. The incidence of vault hematoma after vaginal hysterectomy is variably reported—from approximately 25% to as much as 98% [4–6]. Such disparity in diagnosis may be due to differing definitions of vault hematoma and/or diagnostic modalities. There are many applications of ultrasonography in gynecology, although they are less crucial than in obstetrics. Role of ultrasonography in routine evaluation of post-hysterectomy patients is new and the role is not well defined. Transabdominal sonography may be used to detect, and eventually guide the drainage of fluid collections. The present study focused on vaginal hysterectomy patients having transabdominal ultrasound to find the incidence of postoperative vault hematoma and its correlation with postoperative morbidity.

## 2. MATERIALS AND METHOD

The study was initiated as a prospective-observational study at Education and Research Hospital, during the period of January 2003 to January 2005 where 103 consecutive women who underwent vaginal hysterectomy, with pelvic floor repair operations. Each woman gave consent to the study before the operation. In each case, similar operative techniques were employed by a variety of experienced surgeons. The vaginal vault was closed and not reperitonealised. Women received a single dose of intravenous antibiotics at time of surgery. Amount of blood loss, units of blood transfused, and operating time were recorded for every patient. Postoperative morbidity was assessed by the following parameters which include postoperative fever, drop in hemoglobin (preoperative to third day after operation), and need for blood transfusion (excluding those transfused for preoperative anaemia). Febrile morbidity temperature was >38°C on at least two days after operation. Preoperative anticoagulants did not administer all cases. Each woman had a transabdominal ultrasound examination by a gynecologically trained ultrasonographer, on the first or third postoperative day. The size of any vault collection (nonperistaltic complex echogenic mass) was classified small if its mean diameter was <5 cm, and large if 5 cm or more. It was possible to divide these patients into two groups according to largest diameter of hematoma, to measure the significance of size with respect to postoperative morbidity. The ultrasonographers were not informed of other aspects of the women's postoperative recovery. Staff blinded to the ultrasound findings, documented nursing and medical records. Patients with detected pelvic hematomas were subject to follow-up ultrasound scan at after 7 days. Pelvic hematomas only had vaginal vault hematoma. The patient of large-sized hematoma (>5 cm) was subjected to evacuation of hematoma. Under lithotomy position, with aseptic precautions, without anaesthesia, the central two sutures of chromic catgut were removed, which was followed by digital exploration and evacuation of hematoma. Patients with small-sized hematomas (<5 cm) were observed for spontaneous resolution of the hematoma and development of any complications. All patients were assessed preoperatively for presence of any high-risk factors including hypertension, chronic intake of aspirin, ischemic heart disease, anticoagulant therapy, diabetes mellitus, cerebrovascular disease, coagulation disturbance, chronic obstructive, or restrictive lung disease. The results were subject to x^2^ test to assess significance. But this was not possible due to lack of normal distribution (e.g., hemoglobin drop), the Mann-Whitney U test was applied. All analyses were passed through SPSS software package. A *P* value of <.005 was considered statistically significant.

## 3. RESULTS

The 103 patients who underwent vaginal operations were assessed for various parameters. Twenty (19.4%) had a vaginal vault hematoma (Group 1) and 83 (80%) had no hematoma (Group 2) were detected (Table 1). The largest diameter of the hematoma varied between 2 cm and 9 cm. Mean age 52.32 ± 10.7 and 50.29 ± 6.51 was detected in two groups. No significant difference was observed in the mean operative time that was 95 minutes in patients with hematoma and 110 minutes in those without hematoma. The average drop in hemoglobin was, as anticipated, higher in those women with a hematoma. Eleven percent (12/103) of patients, 40% of the patients with pelvic hematomas, developed postoperative morbidity. Febrile morbidity was evident since 40% (8/20) of patients in Group 1 compared to only 2.4% (2/83) in Group 2 experienced febrile morbidity. The difference was statistically significant. Out of all women having vault hematoma, 70% (14/20) had small-sized hematoma and 30% (6/20) had large-sized hematoma (Table 2). Fifty percent of patients (3/6) with large-sized hematoma as compared to only 25% (5/14) with small-sized hematoma suffered from febrile morbidity. Largest percentage of hematomas belonged to small-sized hematoma and it was about two times larger than the subgroup. Of the 103 women, 4 required preoperative blood transfusion due to either operative bleeding or drop in hemoglobin, or both. One woman in the nonhematoma group required blood transfusion and excessive blood loss was documented at operation, estimated at >500 mL. In the group with a hematoma, three women required transfusions and only two of these had excessive operative bleeding noted. Large-sized hematomas with febril morbidity (3/6) drained vaginally. Small vaginal vault hematomas were managed by no active intervention. Follow-up scans were done weekly. A mean duration of 25 days was required for resolution of the hematoma. None of the patients in either group required readmission to the hospital for any postoperative complications.

## 4. DISCUSSION

A postoperative pelvic hematoma can cause serious morbidity, especially if it is large and becomes infected. Many patients may be asymptomatic; whereas some may present with postoperative bleeding per vaginum (spotting to profuse bleeding per vaginum) postoperative discomfort, abdominal distension, paralytic ileus, continuous fever, foul smelling discharge per vaginum, abscess formation, tenesmus, nausea, vomiting, and diarrhoea. Some authors suggested that the presence of sonographically diagnosed pelvic hematoma is associated with febrile morbidity [7], while others were unable to demonstrate such a relationship [5, 9, 10]. In our study, the overall incidence of vault hematoma was 19.4%, 70% had small-sized hematoma and 30% had large-sized hematoma. In other studies, incidence of postoperative hematoma was detected 25–98% [4, 7]. Differences in surgical technique may be one explanation for this discrepancy, particularly the degree to which the extraperitoneal space adjacent to the vaginal vault is obliterated. In our study, no statistical difference was found in the mean duration of postoperative hospital stay in the two groups. Haines et al. support these findings [10]. The hematoma group was also prone to a greater drop in hemoglobin concentration and spent on average 2.6 days longer in hospital than those with no hematoma. The percentage of hemoglobin drop and the number of patients showing the drop in our study are in total contradiction to other studies [5, 7]. In our study, 40% in patients with vaginal vault hematoma developed postoperative febrile morbidity. Kuhn and de Crespigny, utilizing transabdominal ultrasonography, evaluated 50 consecutive patients following vaginal hysterectomy [4]. There are major differences between their findings and the present study, most notably a claimed incidence of vaginal vault collection of 98% compared with 19.4% in the present study. In the same study, Kuhn also reported a higher incidence of postoperative pyrexia; 70% compared with 11% in the present study [4]. Postoperative febrile morbidity was 16 times more common in subjects with hematoma compared to those without hematoma. Toglia and Pearlman support these findings, in their study 69% of women with postoperative pelvic collection experienced febrile morbidity compared to 12% of those with no collection [8]. In another study, incidence of febrile morbidity was 31% in patients with vaginal vault hematomas [7]. However, other recent studies of small sample populations have concluded that collections of fluid at the vaginal vault following hysterectomy do not contribute to postoperative morbidity. Although Haines and Slavotinek found no significant relation between the detection of vault hematoma and their defined parameters for postoperative morbidity, each study did show a trend towards an increase in febrile morbidity in patients with a hematoma [5, 10]. In another study, there was no correlation between the presence of a collection and indices of postoperative morbidity [8]. Ultrasonography is helpful in detecting and delineating its exact size and location of pelvic hematomas. In the same study, 42% patients had vaginal vault hematoma on sonographic assessment. A review of recent literature reveals that other studies incorporating postoperative transvaginal ultrasound have shown a higher prevalence of vault hematoma following hysterectomy, ranging from 34% to 59% [11]. An extended morbid and complicated postoperative course can be alleviated if the hematoma can be drained. A small penrose drain may be inserted through the drainage tract and left in place for a day or so. If drainage cannot be achieved in this simple way, drainage with guidance of ultrasonography, or if it fails, then using computed tomography or through an abdominal incision may be necessary. If the hematoma can be drained, the patient's recovery will be more prompt. In exceptional cases where drainage may be difficult or contraindicated and infection is not a serious problem, the hematoma may be allowed to gradually resolve over a few months. Unfortunately sometimes, a hematoma will not resolve completely but persists and continues to cause pain. In our study, all the postoperative large hematomas were situated in close proximity to the vaginal vault; hence vaginal drainage was possible. Small vaginal vault hematomas were unlikely to cause postoperative morbidity. They were left alone with watchful expectancy and follow-up ultrasonography for resolution of the hematoma done weekly. This study suggests that asymptomatic pelvic hematomas can be recognized by early postoperative ultrasound scan which are unlikely to be detected clinically. Using postoperative ultrasound, we can identify a population of women at increased risk of postoperative morbidity following vaginal hysterectomy. Large-sized hematoma may develop postoperative morbidity and required vaginal drainage of the hematoma. All patients undergoing vaginal hysterectomy alone do not require undergoing routine postoperative pelvic ultrasound scan.

## Figures and Tables

**Table 1:** Summary of results.

	Hematoma	Nonhematoma
	(*n* = 20)	(*n* = 83)

Age (mean ± SD)	52.32 ± 10.7	50.29 ± 6.51
Febrile morbidity (*n*,%)	8 (40%)	*2 (2.4%)
Cases with Hb drop (*n*,%)	8 (83.33%)	78 (91.49%)
Hb drop (g/dl) (mean ± SD)	2.32 ± 1.42	*1.24 ± 1.23
Hosp. stay day (mean ± SD)	9.42 ± 2.82	*4.24 ± 3.16
Op. time min (mean ± SD)	95 ± 5.12	110 ± 3.24

*P value significantly.

**Table 2:** Relation between postoperative morbidity and size of hematoma.

	Small hematoma	Large hematoma	Nonhematoma

Number	14	6	83
Hb drop (g/dl)	2.8	1.8	1.24 ± 1.23
Hospital-stay (days)	8.5	14	4.24
Febrile morbidity	5 (25%)	3 (50%)	4 (3.8%)
